# Patient Factors Associated With Teledermatology Visit Type and Submission of Photographs During the COVID-19 Pandemic: Cross-sectional Analysis

**DOI:** 10.2196/38694

**Published:** 2022-11-08

**Authors:** Jordan E Lamb, Robert Fitzsimmons, Anjana Sevagamoorthy, Carrie L Kovarik, Daniel B Shin, Junko Takeshita

**Affiliations:** 1 University of Pittsburgh School of Medicine Pittsburgh, PA United States; 2 Department of Dermatology Perelman School of Medicine at the University of Pennsylvania Philadelphia, PA United States; 3 Department of Biostatistics, Epidemiology and Informatics Perelman School of Medicine at the University of Pennsylvania Philadelphia, PA United States

**Keywords:** teledermatology, telemedicine, COVID-19 pandemic, healthcare disparities, photographs, virtual, skin examination, dermatology

## Abstract

**Background:**

The COVID-19 pandemic necessitated the widespread adoption of teledermatology, and this continues to account for a significant proportion of dermatology visits after clinics have reopened for in-person care. Delivery of high-quality teledermatology care requires adequate visualization of the patient’s skin, with photographs being preferred over live video for remote skin examination. It remains unknown which patients face the greatest barriers to participating in a teledermatology visit with photographs.

**Objective:**

The aim of this study was to identify patient characteristics associated with type of telemedicine visit and the factors associated with participating in teledermatology visits with digital photographs versus those without photographs.

**Methods:**

We performed a cross-sectional analysis of the University of Pennsylvania Health System electronic health record data for adult patients who participated in at least 1 teledermatology appointment between March 1, 2020, and June 30, 2020. The primary outcomes were participation in a live-interactive video visit versus a telephone visit and participation in any teledermatology visit with photographs versus one without photographs. Multivariable logistic regression was performed to evaluate the associations between patient characteristics and the primary outcomes.

**Results:**

In total, 5717 unique patients completed at least 1 teledermatology visit during the study period; 68.25% (n=3902) of patients participated in a video visit, and 31.75% (n=1815) participated in a telephone visit. A minority of patients (n=1815, 31.75%) submitted photographs for their video or telephone appointment. Patients who submitted photographs for their teledermatology visit were more likely to be White, have commercial insurance, and live in areas with higher income, better education, and greater access to a computer and high-speed internet (*P*<.001 for all). In adjusted analysis, older age (age group >75 years: odds ratio [OR] 0.60, 95% CI 0.44-0.82), male sex (OR 0.85, 95% CI 0.75-0.97), Black race (OR 0.79, 95% CI 0.65-0.96), and Medicaid insurance (OR 0.81, 95% CI 0.66-0.99) were each associated with lower odds of a patient submitting photographs for their video or telephone visit. Older age (age group >75 years: OR 0.37, 95% CI 0.27-0.50) and Black race (OR 0.82, 95% CI 0.68-0.98) were also associated with lower odds of a patient participating in a video visit versus telephone visit.

**Conclusions:**

Patients who were older, male, or Black, or who had Medicaid insurance were less likely to participate in teledermatology visits with photographs and may be particularly vulnerable to disparities in teledermatology care. Further research is necessary to identify the barriers to patients providing photographs for remote dermatology visits and to develop targeted interventions to facilitate equitable participation in teledermatology care.

## Introduction

The COVID-19 pandemic necessitated the widespread adoption of teledermatology care across the United States [1]. In response to stay-at-home orders and clinic closures, the Centers for Medicare & Medicaid Services expanded access to telehealth services by removing geographic restrictions and adding reimbursement for asynchronous and telephone encounters [2]. This resulted in a major increase in the use of teledermatology services especially during the early pandemic, and teledermatology has continued to account for a significant proportion of dermatology visits even after clinics have reopened for in-person care [3]. Teledermatology providers may deliver care via live-interactive videoconferencing, store-and-forward asynchronous consultations, audio-only visits, or a combination of these modalities [4]. These remote services have the potential to increase access to dermatologic care in underserved areas, deliver care at a lower cost, and mitigate known barriers to in-person visit attendance [5-9].

However, the existing digital divide has raised concerns about the ability of patients to equitably participate in teledermatology care. Reliable internet access and mobile device ownership vary based on patient age and income [10,11]. Additionally, poor technological infrastructure remains a barrier to telemedicine services in rural areas [12]. Previous studies have demonstrated that older adult patients and non–English-speaking patients were less likely to use teledermatology care compared to in-person care during the COVID-19 pandemic [13,14]. Among patients who do participate in teledermatology, older individuals, racial or ethnic minorities, and those with lower income may be more likely to participate in telephone visits compared to live-interactive video visits [15,16].

Given the visual nature of dermatologic examinations, delivery of high-quality teledermatology care requires adequate visualization of the patient’s skin. It can be difficult to properly visualize the skin through live-interactive video, and photographs are preferred for adequate skin evaluation [17]. Previous evaluations of teledermatology care have not assessed whether patients had submitted photographs for their video or telephone visits, which can be an important determinant of the quality of care received. It is unknown which patients face the greatest barriers to participating in teledermatology visits with photographs. Therefore, we aimed to identify patient characteristics associated with telemedicine visit type and the factors associated with participating in a teledermatology visit with and without digital photographs.

## Methods

### Study Design, Data Source, and Study Population

We performed a cross-sectional study of adult patients (≥18 years old) who participated in at least 1 teledermatology appointment between March 1, 2020, and June 30, 2020, using the University of Pennsylvania Health System electronic health record data. During this period of the COVID-19 pandemic, all nonemergent, nonprocedural dermatologic services were provided remotely. Among the small number of patients who participated in more than 1 teledermatology visit during the study period, only the first visit was evaluated.

### Ethical Considerations

This study was determined to be exempt from full review by the institutional review board at the University of Pennsylvania authorized by 45 CFR 46.104, category.

### Outcomes

The primary outcome was teledermatology visit type. We specifically compared participation in a live-interactive video visit versus a telephone visit and participation in any teledermatology visit with photographs versus one without photographs (ie, video or telephone visit with digital photos submitted via the electronic patient portal versus without submission of digital photographs).

### Covariates

Patient characteristics including age, sex, race and ethnicity, primary language, marital status, insurance, electronic patient portal activation status, and visit diagnosis were extracted from the electronic health record. Race and ethnicity were combined and categorized as follows: non-Hispanic White (reference; hereafter referred to as “White”), non-Hispanic Black (hereafter referred to as “Black”), non-Hispanic Asian (hereafter referred to as “Asian”), Hispanic (any race), or non-Hispanic other race. Educational attainment, poverty level, broadband internet access, and computer access were based on the patient’s zip code of residence and obtained from the 2016-2020 American Community Survey 5-year data [18]. Diagnoses associated with teledermatology visits were categorized as follows: inflammatory and autoimmune diseases (including eczema, acne, psoriasis, and other inflammatory skin diseases as well as all autoimmune/immune-mediated skin diseases), benign neoplasms, malignant and premalignant neoplasms, pigmentary disorders, and other dermatologic conditions (including hair or nail disorders, infections, and other diseases not already categorized). The inflammatory and autoimmune diseases category served as the reference diagnosis category. Diagnostic categories were determined based on the distribution and relatedness of individual *International Classification of Disease, Tenth Revision* (ICD-10) codes associated with teledermatology visits included in the study period. Among visits associated with diagnoses across multiple categories, a primary diagnosis was determined based on an estimate of the importance of skin visualization for the diagnosis according to the following hierarchy (from highest to lowest): malignant and premalignant neoplasms, benign neoplasms, inflammatory and autoimmune diseases, pigmentary disorders, and other dermatologic conditions.

### Statistical Analysis

Patient characteristics were summarized using descriptive statistics and compared across telemedicine visit types using Wilcoxon rank sum test for continuous variables and χ^2^ test for categorical variables. Multivariable logistic regression was performed to calculate odds ratios (ORs) and 95% CIs for the associations between patient characteristics and the study outcomes. A purposeful selection modeling approach was used to build the multivariable model [19]. All covariates with significant (*P*<.05) associations with the outcome on bivariate analyses as well as age and sex were included. For the evaluation of teledermatology visits with photographs versus without photographs, electronic patient portal activation status was not included as a covariate in the multivariable model because use of the electronic portal was required to submit photographs for nearly the entirety of the study period. A sensitivity analysis was also performed including only patient-level variables (excluding educational attainment, poverty level, broadband internet access, and computer access) in the multivariable regression models for each outcome of interest. All analyses were performed using SAS 9.4 (SAS Institute Inc).

## Results

In total, 5717 unique patients completed at least 1 teledermatology visit during the study period; 68.25% (n=3902) of these patients had a video visit, and 31.74% (n=1815) had a telephone visit (Table 1). Fewer than one-third of these patients (n=1815, 31.74%) submitted photographs for their video or telephone appointment. The median (IQR) age of the patients was 54 (36-66) years, and most patients were female (n=3712, 64.93%). The racial or ethnic distribution of patients was as follows: 67.73% (n=3872) White, 17.39% (n=994) Black, 3.48% (n=199) Asian, 2.26% (n=129) Hispanic, and 9.15% (n=523) other race. Most patients had commercial insurance (n=2917, 51.02%), and 91.18% (n=5213) had an activated electronic patient portal account that could be used to send messages, documents, or photos electronically to their health care provider. Most visits were associated with a single diagnostic category (n=3349, 58.58%). The most common primary diagnosis category seen during the study period was inflammatory and autoimmune diseases (n=3188, 55.76%), and the most common specific diagnoses were eczema or dermatitis, acne or rosacea, and psoriasis, regardless of visit type.

**Table 1:** Characteristics of patients who completed video versus telephone teledermatology visits.

Characteristic				Overall (N=5717)		Video visits (n=3902)	Telephone visits (n=1815)		*P* value	
Age (years), median (IQR)				54 (36-66)		52 (35-65)	58 (39-70)		<.001	
**Age, n (%)**										
		≤30 years		929 (16.25)		697 (17.86)	232 (12.78)		<.001	
		31-45 years		1260 (22.04)		917 (23.50)	343 (18.90)			
		46-60 years		1374 (24.03)		954 (24.45)	420 (23.14)			
		61-75 years		1537 (26.88)		997 (25.55)	540 (29.75)			
		75+ years		617 (10.79)		337 (8.64)	280 (15.43)			
**Sex, n (%)**										
	Female		3712 (64.93)		2493 (63.89)			1219 (67.16)		.02
	Male		2005 (35.07)		1409 (36.11)			596 (32.84)		
**Race/ethnicity, n (%)**										
	White		3872 (67.73)		2696 (69.09)			1176 (64.79)		<.001
	Black		994 (17.39)		615 (15.76)			379 (20.88)		
	Asian		199 (3.48)		149 (3.82)			50 (2.75)		
	Hispanic		129 (2.26)		90 (2.31)			39 (2.15)		
	Other		523 (9.15)		352 (9.02)			171 (9.42)		
**Primary language, n (%)**										
	English		5641 (98.67)		3852 (98.72)			1789 (98.57)		.89
	Spanish		24 (0.42)		16 (0.41)			8 (0.44)		
	Other		52 (0.91)		34 (0.87)			18 (0.99)		
**Marital status, n (%)**										
	Married/partner		2930 (51.25)		2021 (51.79)			909 (50.08)		.04
	Single		2126 (37.19)		1461 (37.44)			665 (36.64)		
	Divorced/widowed		574 (10.04)		362 (9.28)			212 (11.68)		
	Other		87 (1.52)		58 (1.49)			29 (1.60)		
College graduate (%), median (IQR)				44.20 (27.40-60.0)		45.50 (28.60-61.50)	41.20 (27.10-57.0)		<.001	
Living in poverty (%), median (IQR)				7.00 (4.20-13.40)		6.70 (4.10-13.40)	7.30 (4.70-15.10)		.001	
**Insurance, n (%)**										
	Commercial		2917 (51.02)		2146 (55.00)			771 (42.48)		<.001
	Medicare		1598 (27.95)		956 (24.50)			642 (35.37)		
	Medicaid		740 (12.94)		488 (12.51)			252 (13.88)		
	Other		48 (0.84)		31 (0.79)			17 (0.94)		
	Mixed		344 (6.02)		234 (6.00)			110 (6.06)		
	Missing		70 (1.22)		47 (1.20)			23 (1.27)		
**Electronic patient portal, n (%)**										
	Activated		5213 (91.18)		3657 (93.72)			1556 (85.73)		<.001
	Not Activated		504 (8.82)		245 (6.28)			259 (14.27)		
With broadband internet (%), median (IQR)				89.90 (83.60-93.00)		90.10 (84.40-93.00)	89.40 (83.00-93.00)		<.001	
With a computer (%), median (IQR)				93.80 (90.80-95.80)		93.80 (91.0-95.80)	93.60 (90.40-95.60)		<.001	
**Primary diagnosis category, n (%)**										
	Inflammatory or autoimmune		3188 (55.76)		2123 (54.41)			1065 (58.68)		.01
	Benign		409 (7.15)		289 (7.41)			120 (6.61)		
	Malignant		1501 (26.26)		1072 (27.47)			429 (23.64)		
	Pigmentary disorder		78 (1.36)		58 (1.49)			20 (1.10)		
	Other		541 (9.46)		360 (9.23)			181 (9.97)		
**Number of diagnosis categories, n (%)**										
	1		3349 (58.58)		2212 (56.69)			1137 (62.64)		<.001
	2		1980 (34.63)		1391 (35.65)			589 (32.45)		
	≥3		388 (6.79)		299 (7.66)			89 (4.90)		

### Video Versus Telephone Visits

**Figure 1:**
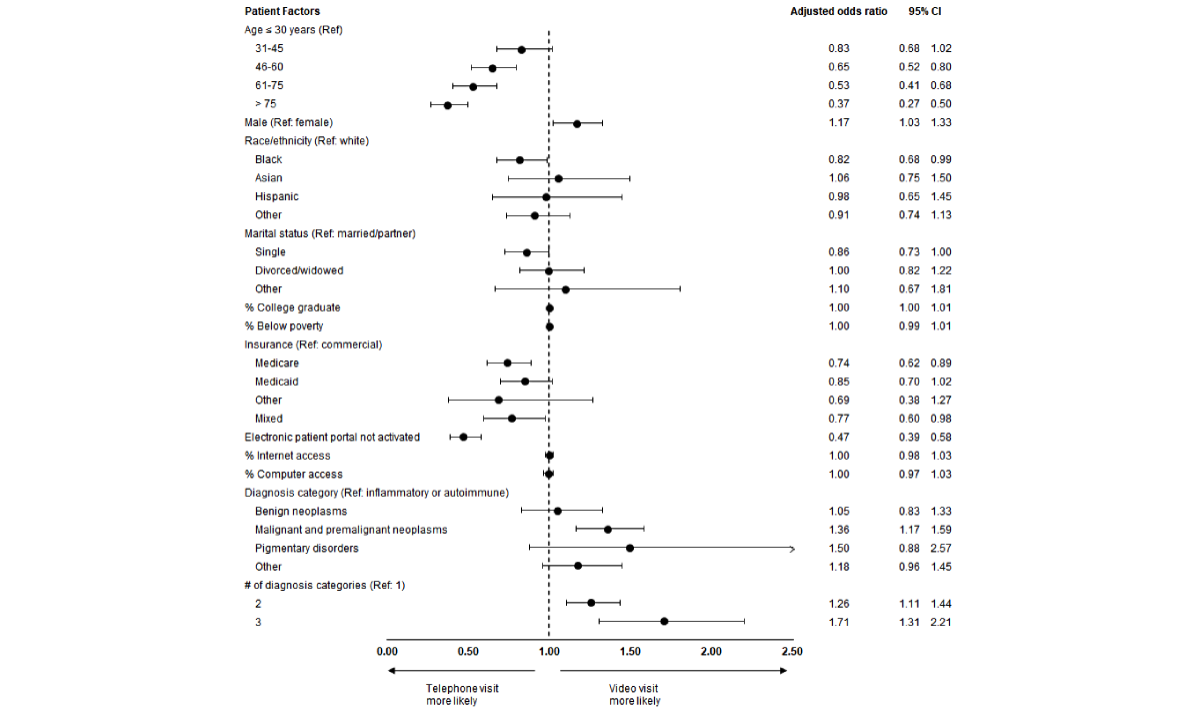
Patient factors associated with video visits compared to telephone visits. Ref: reference group.

In unadjusted analyses, patients who participated in video visits were younger and were more likely to be male, White, and have commercial insurance than were patients who participated in telephone visits; they were also more likely to live in areas with higher income, better education, and greater access to a computer and high-speed internet (Table 1). Notably, patients who participated in video visits were less likely to have Medicare insurance than were those who participated in telephone visits. In adjusted analyses, the following factors were found to be associated with lower odds of a patient participating in a video versus telephone visit: older age (reference age 30 years; age group 46-60 years: odds ratio [OR] 0.65, 95% CI 0.52-0.80; age group 61-75 years: OR 0.53, 95% CI 0.41-0.68; age group >75 years: OR 0.37, 95% CI 0.27-0.50), Black race (OR 0.82, 95% CI 0.68-0.98), Medicare insurance (OR 0.74, 95% CI 0.62-0.89), and nonactivated electronic patient portal account (OR 0.47, 95% CI 0.39-0.58; Figure 1). Male sex (OR 1.17, 95% CI 1.03-1.33), malignant neoplasm primary diagnosis category (OR 1.36, 95% CI 1.17-1.59), and having more than 1 primary diagnosis category (2 categories: OR 1.26, 95% CI 1.11-1.44; 3 categories: OR 1.71, 95% CI 1.31-2.21) were each associated with higher odds of a patient participating in a video versus telephone visit (Figure 1). The results were robust to a sensitivity analysis that included only patient-level variables in the multivariable regression model.

### Visits With Versus Without Photographs

**Figure 2:**
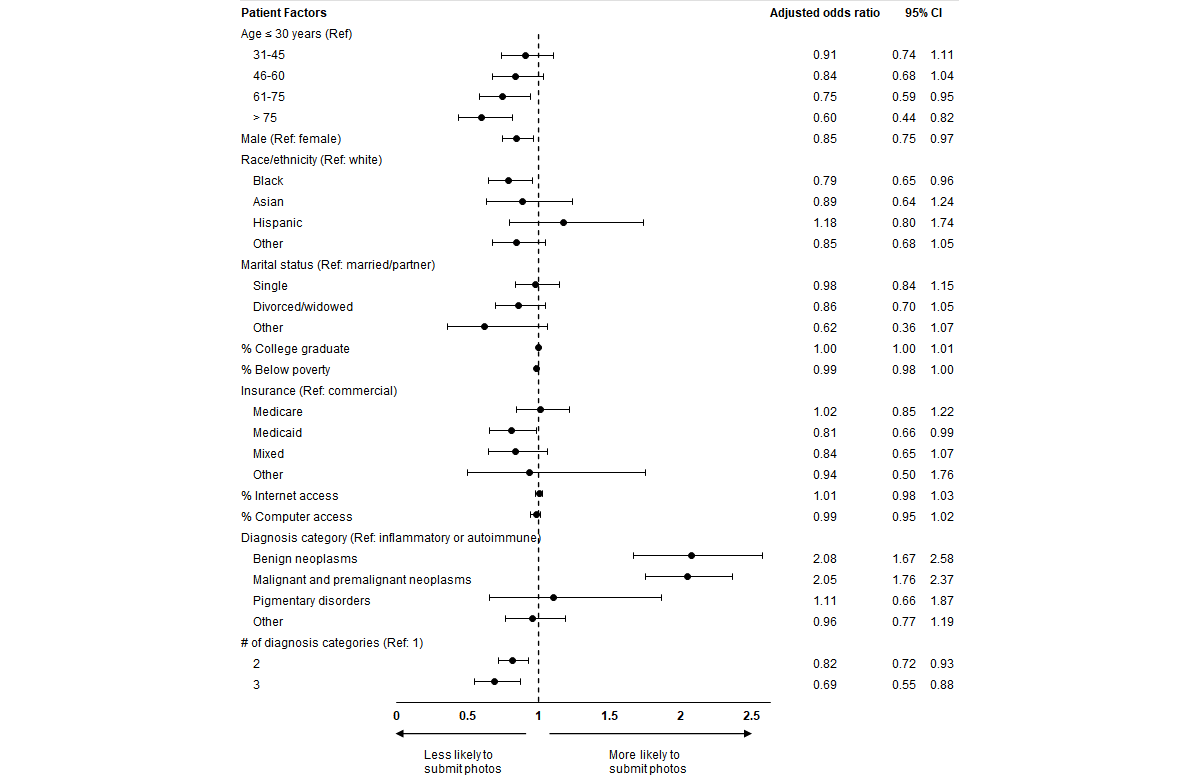
Patient factors associated with submitting photographs for a virtual visit. Ref: reference group.

In unadjusted analyses, there were no significant differences in age or sex between patients who submitted photographs for their video or telephone visit versus those who did not (Table 2). However, patients who submitted photographs for their teledermatology visit were more likely to be White, have commercial insurance, and live in areas with higher levels of income, better education, and greater access to a computer and high-speed internet, than were patients who did not submit photographs (Table 2). Additionally, patients who submitted photographs were less likely to have Medicaid insurance than were those who did not submit photographs. In adjusted analyses, the following factors were found to be associated with lower odds of a patient submitting photographs for their video or telephone visit: older age (reference age 30 years; age group 61-75 years: OR 0.75, 95% CI 0.59-0.95; age group >75 years: OR 0.60, 95% CI 0.44-0.82), male sex (OR 0.85, 95% CI 0.75-0.97), Black race (OR 0.79, 95% CI 0.65-0.96), Medicaid insurance (OR 0.81, 95% CI 0.66-0.99), and more than 1 primary diagnosis category (2 categories: OR 0.82, 95% CI 0.72-0.93; 3 categories: OR 0.69, 95% CI 0.55-0.88). The benign neoplasm (OR 2.08, 95% CI 1.67-2.58) and malignant neoplasm (OR 2.05, 95% CI 1.76-2.37) primary diagnosis categories were each found to be associated with higher odds of a patient submitting photographs for their video or telephone visit (Figure 2). These results were also robust to a sensitivity analysis that included only patient-level variables in the multivariable regression model.

**Table 2:** Characteristics of patients who completed teledermatology visits with photographs versus without photographs.

Characteristic			Visits with photographs (n=1815)	Visits without photographs (n=3902)	*P* value
Age (years), median (IQR)			54 (36-67)	53 (36-66)	.42
**Age, n (%)**					
		≤30 years	292 (16.09)	637 (16.32)	.65
		31-45 years	388 (21.38)	872 (22.35)	
		46-60 years	437 (24.08)	937 (24.01)	
		61-75 years	510 (28.10)	1027 (26.32)	
		75+ years	188 (10.36)	429 (10.99)	
**Sex, n (%)**					
		Female	1190 (65.56)	2522 (64.63)	.49
		Male	625 (34.44)	1380 (35.57)	
**Race/ethnicity, n (%)**					
		White	1344 (74.05)	2528 (64.79)	<.001
		Black	222 (12.23)	772 (19.78)	
		Asian	57 (3.14)	142 (3.64)	
		Hispanic	43 (2.37)	86 (2.20)	
		Other	149 (8.21)	374 (9.58)	
**Primary language, n (%)**					
	English		1798 (99.06)	3843 (98.49)	.14
	Spanish		7 (0.39)	17 (0.44)	
	Other		10 (0.55)	42 (1.08)	
**Marital status, n (%)**					
	Married/partner		999 (55.04)	1931 (49.49)	<.001
	Single		637 (35.10)	1489 (38.16)	
	Divorced/widowed		161 (8.87)	413 (10.58)	
	Other		18 (0.99)	69 (1.77)	
College graduate (%), median (IQR)			49.30 (30.06-62.20)	41.90 (27.40-58.20)	<.001
Living in poverty (%), median (IQR)			6.40 (4.00-10.30)	7.30 (4.70-14.70)	<.001
**Insurance, n (%)**					
	Commercial		976 (53.77)	1941 (49.74)	<.001
	Medicare		514 (28.32)	1084 (27.78)	
	Medicaid		179 (9.86)	561 (14.38)	
	Other		15 (0.83)	33 (0.85)	
	Mixed		103 (5.67)	241 (6.18)	
	Missing		28 (1.54)	42 (1.08)	
**Electronic patient portal, n (%)**					
	Activated		1813 (99.89)	3400 (87.13)	<.001
	Not activated		2 (0.11)	502 (12.87)	
With broadband internet (%), median (IQR)			90.80 (86.40-93.10)	89.60 (83.00-93.00)	<.001
With a computer (%), median (IQR)			94.30 (91.80-96.00)	93.60 (90.40-95.70)	<.001
**Primary diagnosis category, n (%)**					
	Inflammatory or autoimmune		845 (46.56)	2343 (60.05)	<.001
	Benign		178 (9.81)	231 (5.92)	
	Malignant		623 (34.33)	878 (22.50)	
	Pigmentary disorder		20 (1.10)	58 (1.49)	
	Other		149 (8.21)	392 (10.05)	
**Number of diagnosis categories, n (%)**					
	1		1114 (61.38)	2235 (57.28)	.01
	2		581 (32.01)	1399 (35.85)	
	≥3		120 (6.61)	268 (6.87)	

## Discussion

### Principal Findings

In our cross-sectional study of patients who participated in a teledermatology visit during the early COVID-19 pandemic, we found that patients who were older, male, Black, or had Medicaid insurance were less likely to provide photographs for their teledermatology visit after adjusting for sociodemographic factors, diagnosis category, and level of computer and internet access. Patients who were older or Black were also less likely to participate in video visits than in telephone visits. Our study identifies patient populations that may be particularly vulnerable to disparities in teledermatology care.

Previous studies have also found that older patients are more likely to partake in telephone visits than in live-interactive video or asynchronous store-and-forward teledermatology visits [15,16]. Video- and image-based telemedicine encounters require the patient to have access to a smartphone, tablet, or computer; a reliable internet connection; and the ability to follow specific instructions to access and use a telemedicine platform. According to the American Community Survey Reports, people aged 65 years or older have the lowest levels of computer ownership and internet subscriptions [10]. Older adult patients are also more likely to report discomfort with using the internet and have lower electronic health literacy [20]. Many online telemedicine platforms require patients to download a mobile app, sign up for an account, enter demographic information, and memorize a password, all of which can be significant barriers for older patients who may have less experience with this technology or who may be experiencing cognitive decline [21]. Using telemedicine platforms that can be accessed within an existing internet browser, sending direct email or SMS text message links to the appointment, and providing comprehensive appointment instructions ahead of time may help to engage older patients in more teledermatology services [21,22].

In addition to older age, Black race was also associated with a lower likelihood of engaging in a video visit and a lower likelihood of submitting a photograph with any teledermatology visit in this study. A survey of patients’ perceptions of medical photography identified that Black patients reported more discomfort with clinical photography and were less likely to agree that it could enhance care [23]. These beliefs are consistent with low levels of trust in the medical system and concerns about personal privacy that stem from a history of racial discrimination in medicine and research [24], and we must be conscientious of this historical context when asking patients to submit sensitive photographs. Another possible explanation for lower photograph submissions is that Black patients may be less likely to enroll in electronic patient portals [25,26]. During this study period, patient portal enrollment was necessary to submit photographs to dermatologists, and this may also contribute to the differences in photograph submission between Black and White patients after adjusting for internet and computer access levels. More broadly, successful enrollment in the electronic patient portal likely represents a patient’s overall familiarity with and frequency of technology use, both of which contribute to patients’ willingness to participate in video visits [27]. Additionally, the racial differences we have observed likely represent other socioeconomic and infrastructural barriers, such as stable internet connection and personal smartphone or tablet ownership, that were not directly measurable in our study and prevent equitable participation in teledermatology care.

We also identified that having Medicaid insurance was independently associated with a lower likelihood of participating in a teledermatology visit with a photograph. This gap may be the result of decreased access to the technology necessary to capture and submit high-quality photographs to a health care provider. Around one-quarter of adults with household income below US $30,000 report they do not own a smartphone, whereas smartphone ownership is nearly 100% among adults in households earning US $100,000 or more a year [11]. To overcome this financial barrier to remote care, access to a mobile device may need to be considered a medical necessity for low-income, geographically isolated patients [28].

Lastly, there were sex differences in type of teledermatology care received in this study. Prior to the COVID-19 pandemic, research showed that female patients were more likely than males to choose telemedicine visits over in-person appointments [29]. Here, we found that female patients were more likely to participate in telephone visits compared to video visits. This tendency has also been observed in the outpatient cardiology setting [30], while conversely, a study of telemedicine visits in an interventional radiology clinic found that female patients were more likely to complete video visits compared to telephone visits [31]. In our study, females were less likely to use video visits, while they were more likely to submit photographs with their video or telephone appointments. Further investigation is needed to better characterize these sex differences and understand patient preferences for telemedicine modalities.

This study is novel in that we were able to identify patient factors associated with skin visualization with photographs for teledermatology visits. Technologic advances in the resolution of digital photography have made skin visualization through photographs superior to video [32]. Although there are no known studies that directly measure the quality of teledermatology care between audio- or video-only visits to those with photographs, proper visualization of the skin is necessary for dermatologic examination. A recent survey showed that most dermatologists felt that telemedicine video quality was insufficient to provide care equivalent to an in-person visit and that uploading high-quality photographs was needed to supplement the video [33,34]. Therefore, patients relying on audio- or video-only teledermatology may be vulnerable to receiving lower-quality care than those seen via a hybrid method with photographs.

The COVID-19 pandemic has highlighted the longstanding structural inequities that exist in the United States that result in health and health care disparities. Certain populations have been disproportionately affected by COVID-19. For example, Black Americans have a higher burden of disease incidence, hospitalizations, and deaths due to COVID-19 [35,36]. Additionally, patients with public insurance are more likely to be admitted to the hospital for COVID-19–related complications [37]. As we adapt to the ongoing effects of the pandemic, we must ensure that practice changes do not further exacerbate existing disparities in access to and use of health care services. Policies that expand telemedicine access should also be coupled with strategies to broaden access to reliable and high-speed internet and the technological devices needed to participate in high-quality remote care. More funding is needed at the state and national levels to support growing technological infrastructure as are community interventions to promote electronic health literacy and provide access to publicly available electronic resources.

### Limitations

This study has several limitations. The data were collected from a single, urban, academic medical center, which may limit the generalizability of the results. We did not have access to patient-level information for income, education, or computer and internet access, so these characteristics were estimated based on each patient’s zip code of residence. We did not assess the quality of the photographs provided for the visits, and additionally, we were unable to assess differences in the quality of care received or dermatologic outcomes between visits with video, photograph, or no direct skin visualization. Direct visualization of the skin may not be necessary for all dermatology visits, particularly for established patients with stable, chronic conditions. We were unable to completely account for the reason for visit, which may impact how necessary it is to have skin visualization through video or photographs. Future studies are needed to determine if the modality and quality of skin visualization during a teledermatology appointment impact diagnostic accuracy, need for in-person follow-up visits, or specific skin health outcomes.

### Conclusions

This study provides evidence that patients who are older, male, Black, or who have Medicaid insurance are less likely to participate in a teledermatology visit with photographs. Inadequate skin visualization during the virtual dermatologic examination may make this population particularly vulnerable to disparities in teledermatology care. As telemedicine continues to be an integral part of dermatology care delivery after the COVID-19 pandemic, further research is necessary to identify the barriers to sending photographs and to develop targeted interventions to facilitate equitable participation in teledermatology visits.

## Abbreviations

OR: odds ratio.
